# Long-Acting Extracellular Vesicle-Based Biologics in Osteoarthritis Immunotherapy

**DOI:** 10.3390/bioengineering12050525

**Published:** 2025-05-15

**Authors:** Philip Drohat, Max Baron, Lee D. Kaplan, Thomas M. Best, Dimitrios Kouroupis

**Affiliations:** 1 Department of Orthopedics, UHealth Sports Medicine Institute, Miller School of Medicine, University of Miami, Miami, FL 33146, USA; pdrohat@med.miami.edu (P.D.); mcb224@med.miami.edu (M.B.); kaplan@med.miami.edu (L.D.K.); txb440@med.miami.edu (T.M.B.); 2 Diabetes Research Institute, Cell Transplant Center, Miller School of Medicine, University of Miami, Miami, FL 33136, USA

**Keywords:** osteoarthritis, extracellular vesicles, biologic therapy, immunomodulation

## Abstract

Osteoarthritis (OA) is a chronic degenerative joint disease characterized by low-grade inflammation, cartilage breakdown, and persistent pain. Despite its prevalence, current therapeutic strategies primarily focus on symptom management rather than modifying disease progression. Monoclonal antibodies and cytokine inhibitors targeting inflammatory pathways, including TNF-α and IL-1, have shown promise but remain limited by inconsistent efficacy and safety concerns. Long-acting biologic therapies—ranging from extended-release formulations, such as monoclonal antibodies and cytokine inhibitors, to gene therapy approaches—have emerged as promising strategies to enhance treatment durability and improve patient outcomes. Extracellular vesicles (EVs) have gained particular attention as a novel delivery platform due to their inherent stability, biocompatibility, and ability to transport therapeutic cargo, including biologics and immunomodulatory agents, directly to joint tissues. This review explores the evolving role of EVs in OA treatment, highlighting their ability to extend drug half-life, improve targeting, and modulate inflammatory responses. Additionally, strategies for EV engineering, including endogenous and exogenous cargo loading, genetic modifications, and biomaterial-based delivery systems, are discussed.

## 1. Introduction

### 1.1. Osteoarthritis Pathophysiology

Osteoarthritis (OA), traditionally considered a non-inflammatory type of arthritis primarily due to mechanical overloading of the joint and periarticular tissues, has been increasingly recognized to also involve an imbalance between pro-inflammatory and anti-inflammatory cytokines [1]. Multiple OA phenotype classifications have been proposed to account for disease heterogeneity [2,3,4,5,6]. Regardless of the phenotype, patients typically have similar clinical endpoints, pain, and activation of low-grade joint inflammatory/immune cascades [2,3,4,5,7]. Specifically, the imbalance between pro- and anti-inflammatory signaling contributes to the chronic, low-grade inflammation often observed in patients with OA [8]. Two major inflammatory cytokines, interleukin-1 (IL-1) and tumor necrosis factor-alpha (TNF-alpha), both produced by the synovium and infrapatellar fat pad (IFP), are notably involved in the onset and progression of OA, with extracellular vesicles (EVs) playing a key role in transporting these cytokines within joint tissues [1,9]. Dysfunctional chondrocytes further exacerbate the condition by disrupting the balance of cartilage-degrading enzymes such as matrix metalloproteinase-13 (MMP-13) and ADAMTS5, leading to cartilage breakdown and disease progression [10].

In patients with knee OA (KOA), there can be further contribution to disease pathology from both the IFP and the synovium. Both players contribute to immune system dysregulation in OA, helping to create a chronic state of local inflammation. The IFP specifically is responsible for the secretion of pro-inflammatory molecules, including IL-1β and matrix metalloproteases, as well as hormones, into the synovium. Leptin is a hormone directly responsible for the activation of pro-inflammatory M1 macrophages in the IFP. Other cytokines, including IL-6 and IL-8, as well as prostaglandins, help to recruit immune cells to the IFP and the synovium. Immune cells are also recruited to the synovium via substance P (SP), a neuropeptide that facilitates the extravasation of these cells [11]. Specifically, the role of SP in the development of OA was demonstrated in a recent study exploring the therapeutic effects of EVs generated from IFP mesenchymal stem/stromal cells (MSC), which expressed CD10 (neprilysin), a neutral endopeptidase that degrades SP [12]. In this study, CD10High and CD10Low EVs were found to have unique immunomodulatory and anabolic miRNA contents. However, CD10High EVs exhibited potent chondroprotective and anabolic downstream effects, resulting in reduced cartilage degeneration and synoviocyte inflammation [12]. These findings highlight the role SP plays in OA pathophysiology as well as the potential role of infrapatellar fat pad-derived mesenchymal stem cells (IFP-MSC) EVs in OA therapeutics.

OA remains the most prevalent subtype of arthritis, yet current therapeutic options focus primarily on pain and symptom management rather than addressing the underlying disease mechanisms [13]. Without therapeutic agents that interfere with the progression of the disease, individuals are frequently left with one option: joint replacement. Accordingly, there is an unmet need for research into novel disease-modifying agents or innovative drug delivery methods that address the underlying disease process, as well as their translation to the market and patient care. Given the role of inflammation in OA pathogenesis, targeted approaches to modulate these pathways hold promise for improving outcomes in individuals possessing this phenotype.

### 1.2. Monoclonal Antibodies and Cytokine Inhibitors in OA

Monoclonal antibodies have emerged as promising candidates for the treatment of OA by targeting specific pathways involved in pain and disease progression (Table 1). Among these, infliximab and adalimumab, both TNF-α inhibitors, have demonstrated effectiveness in reducing pain, with adalimumab showing particular efficacy when administered intra-articularly (IA) [8,14]. Interestingly, in studies focusing solely on hand OA, these inhibitors led to improved tissue structure, including reduced evidence of radiographic disease progression, as well as fewer bone marrow lesions and erosive soft tissue swelling; however, they did not yield benefits in terms of symptom relief specifically related to hand OA [15].

Similarly, monoclonal antibodies targeting nerve growth factor (NGF) have shown significant potential in pain management. Tanezumab has proven effective in relieving pain associated with hip and knee OA that was previously unresponsive to non-opioid medications, and repeated injections of Tanezumab provide pain relief for up to 56 weeks [16,17,18]. Fanisumab, another NGF inhibitor, has undergone multiple phase IIb/III clinical trials, where it demonstrated pain reduction and improved function, as measured by the Western Ontario and McMaster Universities Osteoarthritis index (WOMAC) and patient global assessment (PGA) scores, in patients with moderate to severe hip or knee OA [13,19]. Additionally, in patients with moderate to severe knee or hip OA, Fulranumab has shown comparable efficacy to oxycodone in a phase II trial with further validation in a phase III study through subcutaneous administration, resulting in reduced pain and enhanced function, as measured by WOMAC and PGA scores [20,21]. While NGF is a promising target for OA therapy, monoclonal antibodies targeting NGF raise questions about their safety profile, with adverse events including rapidly progressive OA, abnormal peripheral sensation, and cardiac or gastrointestinal events, reported [22]. 

Bevacizumab, a monoclonal antibody targeting vascular endothelial growth factor (VEGF), has demonstrated safety when administered into the knees of rabbits with osteoarthritis (OA) [23,24]. This approach reduces joint inflammation, synovial proliferation, and cartilage degradation. Moreover, IA administration was more effective and safer than systemic delivery [25]. Of the monoclonal antibodies studied for OA, bevacizumab was the most promising due to its ability to slow and potentially halt the progression of OA in animal models [26].

Monoclonal antibodies targeting interleukin pathways have also been explored. Canakinumab, an IL-1 beta inhibitor, reduced the need for hip or knee arthroplasty, suggesting its potential as a disease-modifying therapy [27]. AMG 108, a monoclonal antibody targeting the IL-1 receptor, was explored as a potential means of providing pain control in knee OA, but did not produce significant findings [28]. Another innovative approach involves M6495, a monoclonal antibody targeting ADAMTS5, which is currently under investigation for its potential role in treating OA [29].

While monoclonal antibodies may play a role in future OA therapeutics, numerous challenges remain. Among the monoclonal antibodies studied to date, there is considerable variance in both the number of injections and the total amount required. The average number of doses needed was 4.6 ± 3.2, and doses ranged from 0.02 mg/mL to 50 mg/mL [26]. This highlights the lack of standardized procedures and methods for assessing the efficacy of monoclonal antibodies in OA [26].

Some anti-inflammatory biologics have been successfully employed in patients with rheumatoid arthritis (RA), but their application in OA has shown less efficacy [8]. Among these, IL-1 has been identified as a key cytokine upregulated in OA, making it a potential target for biologic therapies [1]. Anakinra, an IL-1 receptor antagonist used to treat both RA and OA, was studied extensively but did not show significant improvement in OA symptoms compared to placebo [16,30].

Further studies on IL-1-targeted therapies have revealed varied outcomes (Table 1) [31]. IL-1 antibodies and IL-1 inhibitors have demonstrated promising results in reducing OA symptoms and enhancing joint functionality, as indicated by the mean change in WOMAC and KOOS functionality scores [31]. However, IL-1 receptor antibodies did not yield the same success. Additionally, while IL-1 antibodies and receptor antibodies were deemed safe, safety concerns arose with the use of IL-1 inhibitors [31]. IL-1 inhibitors have been associated with higher rates of adverse events, the most common being pain, respiratory illness, skin disorders, and gastrointestinal disorders [31].

### 1.3. Long-Acting Therapy and OA

Long-term management of OA as a chronic disease poses significant challenges for patients. IA injectables have been used to provide some pain relief; however, the size of the joint being treated limits the amount of medication that can be injected [32]. This, in combination with the microenvironment of the joint, contributes to the rapid clearance of injectable IA medications [32]. To address this, researchers have explored innovative drug delivery systems to enhance the efficacy and duration of treatments.

One study developed a nanogel designed to extend the release of kartogenin, a small bioactive molecule known for its ability to promote the differentiation of MSC into chondrocytes [32]. Nanogels are hydrophilic polymer networks capable of swelling in aqueous environments to encapsulate and release therapeutic agents in a controlled, stimulus-responsive manner. They are especially helpful in targeted drug delivery and tissue engineering. In the study by Sun et al., a hyaluronic acid-based nanogel was used due to its hydrophilic characteristics [32]. Similarly, another study investigated the use of chitosan-derived biodegradable microspheres to develop a longer-acting intra-articular formulation of lornoxicam, a medication used for pain relief [33]. FX006, an extended-release IA triamcinolone delivered by poly(lactic-co-glycolic acid) (PLGA) microspheres, has undergone phase III studies and was found to successfully prolong pain relief compared to the instant-release formulation [18,34,35]. Interestingly, the liposomal formulation of dexamethasone sodium phosphate (DSP), TLC599, demonstrates prolonged drug retention in the joint due to the inherent characteristics of the nanoparticle liposome, as compared to larger microspheres. Phase IIa and III clinical trials of TLC599 demonstrated pain reduction and improved function, as measured by the WOMAC score, for up to 52 weeks [18,36].

Other long-acting therapies for OA that are not dependent on delivery vehicles, such as nanogels, are also being studied. XT-150 is a DNA plasmid that contains a long-acting variant of human IL-10. The medication, which is injected IA, allows for the plasmid to be taken up by synoviocytes, causing them to manufacture the long-acting variant IL-10, which is able to reduce inflammation in KOA [18]. Two phase 1 studies for XT-150 have demonstrated an acceptable safety profile, as well as improved pain and physical function, as measured by the WOMAC score, in patients with KOA for up to 6 months [37].

Two notable clinical trials investigating IA injection of stem cells for the therapy of OA are ongoing in Australia. One study examines the use of MSC-derived integrin α10β1 (XSTEM-OA), specifically a single intraarticular injection of allogeneic X-STEM-OA into the knee joint [Trial ID: NCT05344157]. The other study investigates MSC-derived induced pluripotent stem cells (iPSC). The goals of these studies are to establish efficacy and safety, and to determine the ability to deliver long-term OA relief and potential alterations in disease progression [18,38]. A follow-up study from Spain found that the injection of autologous MSC into the knee joint in patients with OA provided durable pain relief and objective cartilage improvement at two years [39]. A study in China investigating the use of autologous human adipose-derived MSC (haMSC) for KOA found that at 96 weeks, there were improvements in pain, function, and cartilage volume, with the greatest effect in the highest dose group of 5 × 10^7^ cells [40]. Another study out of China investigated human adipose-derived mesenchymal progenitor cells (haMPC) for the treatment of KOA. At 12 month follow up, it was observed that treatment haMPC enhanced joint function as indicated by WOMAC functional score, pain, quality of life, and cartilage regeneration [41].

## 2. EVs as an Emerging Therapeutic Strategy

### 2.1. Overview of EVs

EVs are small, cell-derived vesicles that contain identical internal characteristics (nucleic acid, proteins, and lipids) as their parental cell, which can be categorized into three main types based on size: exosomes, microvesicles, and apoptotic bodies [42]. Exosomes originate from the endosomal system via multivesicular body fusion with the plasma membrane, while microvesicles form by direct plasma membrane budding, leading to distinct molecular compositions [42]. However, current extracellular vesicle preparation methods do not enable the complete isolation of exosomes from other types of vesicles [42]. EVs serve as an umbrella term for vesicles that can either be native, derived directly from the parent cell, or engineered for specific purposes. They function by delivering and releasing their internal cargo or interacting with the cell surface to trigger downstream effects on target cells [43]. EVs are naturally designed to carry materials such as proteins and nucleic acids, offering protection through lipid encapsulation and homing capabilities [44].

Among the types of EVs, exosomes have gained significant attention in the scientific community due to their wide-ranging applications. They hold promise as biomarkers for disease diagnosis, as immunomodulators for activating or suppressing the immune system, and as delivery vehicles for therapeutics [45]. These properties make them particularly relevant to OA therapy, where their delivery and immunomodulatory potential could be exploited for therapeutic benefit. Unlike traditional drug delivery vehicles such as liposomes, which rely on passive accumulation, EVs possess intrinsic targeting capabilities tied to their parental cell origins or engineered modifications [46]. This targeting ability, coupled with their natural function as carriers of nucleic acids and proteins, underscores their potential as a therapeutic delivery system [43].

### 2.2. Extended Duration and Effectiveness of Biologics When Combined with EV Technology

EVs are highly favorable as drug delivery vehicles due to their lipid bilayer, which protects the cargo from degradation, enhances stability in vivo, and prolongs blood circulation time [47]. Additionally, EVs allow for increased drug concentration in target tissues, generating a more effective delivery than free drugs [48]. EVs have been studied as drug delivery vehicles for numerous classes of therapeutics. They have been explored for chemotherapy delivery, where they enable targeted delivery of drugs to cancer cells, reducing systemic toxicity and enhancing therapeutic outcomes. For example, pancreatic cancer cell-derived EVs loaded with Paclitaxel significantly reduced tumor size compared to free (without EVs) Paclitaxel treatments [49]. Moreover, EVs have shown promise in addressing infectious diseases and immune-related conditions by effectively delivering therapeutic agents. Macrophage-derived exosomes loaded with linezolid demonstrated superior efficacy against intracellular MRSA infections compared to free linezolid [50]. Similarly, gingival MSC-derived EVs loaded with antibiotics not only responded to bacterial biofilms but also promoted the regeneration of damaged periodontal tissue, highlighting the dual therapeutic and regenerative potential of EVs [51].

**Figure 1:**
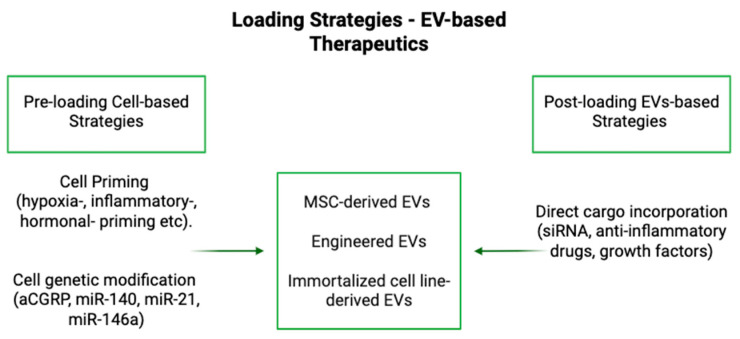
Schematic representation of EV-based therapies. Different EV types—MSC-derived, engineered, and immortalized cell line EVs—contribute to immunomodulation, cartilage protection, and long-acting effects. Pre-loading (genetic modification, cell stimulation) and post-loading (cargo incorporation) strategies enhance EV therapeutic potential.

While the application of EVs as a therapeutic delivery system is broad, a particular area of interest is their use to deliver cytokine-based biologic therapies (Figure 1). EVs have been engineered to carry monoclonal antibodies for anticancer therapy, improving their targeting efficacy against tumor tissues [52,53]. Modifications to the surface of EVs to incorporate monoclonal antibodies have introduced CAR T-cell mimicking therapies, thereby advancing the potential for immunotherapy [52]. Interleukin-10 (IL-10) encapsulated in EVs showed increased stability and precise targeting in the treatment of acute kidney injury (AKI) [54]. In studies of acute respiratory distress syndrome (ARDS), EVs carrying IL-4 and IL-10 payloads reduced inflammation by decreasing pro-inflammatory cytokines such as IL-1 beta and TNF alpha while increasing anti-inflammatory metabolites [55].

With respect to OA and other degenerative joint diseases, some studies have employed EVs for the delivery of therapeutics. EVs loaded with kartogenin have overcome the molecule’s previous limitations related to low water solubility [56]. Studies have demonstrated increased kartogenin concentrations inside cells, both in vitro and in vivo, when delivered via EVs [56]. While EVs can modify the effects of their payload, the cargo itself may also be utilized to adjust the intrinsic therapeutic properties of EVs. The functionality of EVs can be enhanced by modifying their specific cargo or treating them with immunosuppressive cytokines like IL-10, which boost their anti-inflammatory and chondroprotective effects [57].

**Figure 2:**
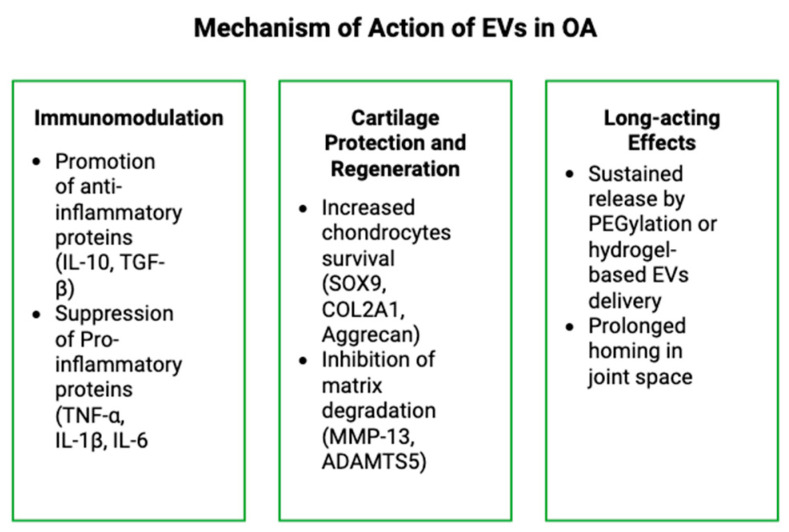
Schematic representation of the mechanism of action of EVs in OA.

Recent advancements in EV engineering have introduced promising strategies to further enhance their therapeutic efficacy in OA (Figure 1 and Figure 2). One such approach involves genetic modification of IFP-MSCs to express an antagonist peptide targeting calcitonin gene-related peptide (aCGRP), a key neuropeptide involved in pain transmission and inflammation [58]. EVs derived from these genetically modified MSCs have demonstrated potent immunomodulatory and analgesic properties, including the ability to modulate macrophage polarization and alter neuronal pain signaling pathways [58]. Additionally, hormonal priming strategies, such as oxytocin exposure during inflammatory conditioning, have been shown to enhance MSC immunosuppressive and regenerative capacities [59]. This approach promotes M2 macrophage polarization and augments the anti-inflammatory effects of EVs, further optimizing their therapeutic potential in OA [59].

## 3. Formulation of EVs to Be Loaded with Specific Attributes

### 3.1. Loading

EVs can be modified using two primary approaches: endogenous and exogenous loading (Table 2) [60,61]. Endogenous loading involves manipulating donor cells to influence the composition of EVs during their biogenesis, or before the EVs are harvested, which can also be described as “preloading” [42,60]. This can be achieved through biochemical methods, such as co-incubation, transfection, or exposure to hypoxic conditions, as well as mechanical methods, including the application of mechanical stress or the utilization of three-dimensional co-culturing techniques [60]. Drugs can be packaged into EVs via endocytosis or receptor-mediated processes, which are influenced by intracellular factors such as cellular stress or signaling pathways [61]. Genetic engineering of donor cells further optimizes this process by enabling the production of specific therapeutic cargos that become encapsulated into EVs during their formation, which can also be accomplished via overexpression of the desired cargo in the parent cell [61,62]. This method allows for greater control over the composition and therapeutic potential of secreted EVs [61].

On the other hand, exogenous loading focuses on modifying EVs directly after their isolation, which can also be described as “post-loading” [42]. Exogenous loading encompasses a variety of techniques for incorporating therapeutic or genetic material into EVs. Incubation involves simple mixing, where drugs diffuse into EVs; however, the efficiency of this process varies depending on factors such as the drug type, incubation time, and experimental conditions [60,61,63]. Ultrasonic waves can also be employed to temporarily disrupt EV membranes, enabling drug entry with high efficiency, although this method poses a risk of structural alterations [61]. Electroporation is another effective technique, using electric fields to permeabilize membranes and facilitate drug loading, with parameters tuned for optimal performance [61,63]. Chemical methods, such as surface functionalization or membrane reconstitution, enable EVs to be chemically modified for improved drug interactions [61]. These post-loaded EVs can be genetically engineered to enhance drug delivery by altering the surface or cargo composition of EVs, enabling precise targeting and therapeutic efficacy [60,61].

Within endogenous or exogenous techniques, there are both active and passive forms of loading. Passive loading relies on incubating EVs with therapeutic compounds, while active loading employs mechanical or chemical strategies to enhance cargo entry [63]. Examples of passive loading include incubation or endocytosis, whereas active loading can take the form of extrapolation, sonication, freeze-thaw, transfection, or numerous other approaches; of note, transfection is a method used in exogenous and endogenous loading [63]. Both methods offer distinct advantages, with the choice of technique depending on the type of cargo and the intended application.

There are countless combinations of possible EVs and their respective cargo, such as exosomes derived from MSC or other donor cells that have been genetically engineered to express therapeutic genes [64]. Genetic engineering is one method used for loading EVs that has been extensively investigated. In our previous study, IFP-MSC transduced with an adeno-associated virus (AAV) vector carrying a gene for a Calcitonin Gene-Related Peptide (CGRP) antagonist generated EVs enriched with 147 unique microRNAs (miRNAs) targeting pathways involved in pain, inflammation, and cartilage homeostasis. These findings indicate that EVs could serve as vehicles for both targeting CGRP and delivering analgesic cargo, offering a multifaceted approach to OA treatment [58]. Chondrocyte affinity protein (CAP) exosomes transported miR-140 beyond the superficial articular cartilage via the mesochondrium, which effectively reduced cartilage degradation and slowed progression of OA in rat models [65]. This further highlights how EVs can be utilized to enhance cell-free immunotherapies [66]. A separate study also utilized CAP exosomes to deliver antisense oligonucleotides targeting MMP13 (ASO-MMP13), which helped to optimize drug delivery as well as prolong retention of ASO-MMP13 in the joint [67]. Another study loaded exogenous miR-223 into EVs derived from human umbilical cord MSC. These engineered EVs were found to create enhanced targeting and delivery of mRNA-223, which maximized the downstream effects of inhibiting inflammasome activation and chondrocyte pyroptosis [68].

Additionally, genetic engineering of MSC has been shown to influence the properties of their derived sEVs, improving their immunosuppressive and chondroprotective capacities [57]. Gene therapy applications have also benefited from EV technology, as demonstrated by hybrid exosomes fitted with chondrocyte affinity peptides and loaded with CRISPR/Cas9-based fibroblast growth factor 18 (FGF18) gene editing tools. Encapsulated in methacrylic anhydride-modified hyaluronic (HAMA) hydrogel, these exosomes promoted cartilage regeneration and reduced ECM degradation, showcasing the potential of utilizing genetic engineering in the loading of EV-based approaches in regenerative medicine [69].

While the combination of parent cell and desired cargo is important, other factors can modify the loading process as well as the final function of EVs. Protein loading into EVs can be augmented through associations with plasma membranes and lipid rafts, in which these lipids improve the efficiency of loading EVs and enhance targeting capabilities [70]. Modifying EV membranes through protein fusion enhances targeting efficiency, although it may interfere with the functional properties of the cargo [56]. Furthermore, hydrophobic compounds can modify the loading process, as they have an increased likelihood of crossing the EV membrane, making them favorable for the loading process efficiency [71]. 

### 3.2. Properties and Modifications of EVs That Generate Specific Functions Unrelated to Cargo Effects

Modifications of EVs as a vehicle, rather than their cargo, obtained by altering the parental cell, offer promise for optimizing their payload efficacy and intrinsic therapeutic properties. Cell-derived vesicles have shown significant potential in the treatment of OA due to their chondroprotective and immunomodulatory properties. EVs derived from embryonic stem cell-induced MSC (ESC-MSC) in the presence of IL-1 beta were found to reduce cartilage destruction and matrix degradation by enhancing collagen type II synthesis and decreasing ADAMTS5 expression [72]. Similarly, CD10 high sEVs demonstrated robust chondroprotective and immunomodulatory effects both in vitro and in vivo, suggesting their potential as a cell-free alternative, particularly in OA phenotypes characterized by synovitis and IFP fibrosis [12]. Additionally, bone marrow-derived MSC (BMSC) exosomes were shown to counteract the inhibitory effects of IL-1 beta in a rat model of OA through the upregulation of COL2A1 and downregulation of MMP13, leading to improved cartilage repair and ECM synthesis [73].

As discussed previously, modification of exosomes with CAP has been shown to enhance the targeted delivery of EV payloads to the joint and increase retention time within the joint. CAP-exosomes, when compared to exosomes that were not ligated with CAP, decreased IL-1 beta-induced chondrocyte damage, as well as suppression of the IL-17 and TNF pathways, leading to reduced inflammation [67]. Modifications to exosomes as delivery vehicles can be enhanced by improving their targeting and stability, while also generating secondary anti-inflammatory effects, which may be beneficial in the treatment of OA [65,67].

The regenerative potential of EVs extends beyond OA treatment to bone repair and tissue regeneration. For instance, MSC genetically modified with bone morphogenetic protein 2 (BMP2) were transformed into EV-producing “factories”, generating exosomes that specifically targeted injured bone sites; these exosomes exhibited enhanced bone regeneration capabilities, demonstrating the potential of engineered EVs in skeletal repair [74]. Furthermore, innovative delivery systems, such as three-dimensional biomaterials and scaffolds, have been employed to improve EV retention and ensure sustained local release, which address the short half-life of EVs caused by rapid cellular uptake and enhance their therapeutic impact [75]. PEGylation is another approach that can be used to achieve a longer half-life of EVs in circulation. Other techniques to achieve the same goal include membrane-bound complement regulators, such as CD55 and CD59, or CD47, which serve as a signal to evade phagocytosis [76]. EVs can be engineered to carry a greater concentration of cargo or be coated with specific cell-targeting ligands to enhance the therapeutic effects of their payload [75,77].

## 4. Considerations for the Use of EVs

### 4.1. Biocompatibility, Stability, and Delivery of EVs 

EVs are easily modified and have demonstrated good stability for long-distance transport, making them highly favorable candidates as drug delivery vehicles [45]. Among EVs, exosomes are the most extensively studied. However, apoptotic vesicles (apoVs) have shown potential as a promising alternative for future OA therapies, which highlights the versatility of EV subtypes and their expanding role in clinical treatments [9]. One of the primary advantages of EVs is their natural encapsulation, which provides low immunogenicity and therefore high biocompatibility as a delivery vehicle [9]. Their surfaces can be bioengineered to display high-affinity targeting domains, allowing for receptor-specific binding to designated tissues [62]. This adaptability enhances their ability to deliver therapeutic cargo precisely and efficiently, minimizing off-target effects and improving treatment outcomes. Furthermore, EVs that express endogenous CD47 demonstrate an extended half-life in circulation and evade macrophage phagocytosis, further enhancing their therapeutic potential [44].

IA injections remain a common method of delivering EVs for joint-related therapies. However, EV-loaded hydrogels are gaining attention as an innovative alternative. Hydrogels offer several advantages, including their compatibility with three-dimensional bioprinting, a reduced risk of rapid cargo clearance, and minimized disruption to cartilage. Additionally, hydrogels enable EVs to penetrate deeper layers, reaching chondrocytes and subchondral bone-resident cells that might otherwise be inaccessible through traditional IA injections [78]. The development of three-dimensional printed scaffolds further enhances EV applications by improving joint homeostasis and offering greater control over release behavior, ensuring a sustained therapeutic effect [79]. By leveraging the natural properties of EVs and engineering their surfaces for targeted delivery, researchers can ideally develop more effective and efficient therapies for OA.

### 4.2. Diffusion

Innovations in delivery systems for EVs have shown promise in improving their retention and effectiveness in cartilage repair. An injectable Diels-Alder crosslinked hyaluronic acid/polyethylene glycol (PEG) hydrogel was designed for MSC-derived sEVs, which demonstrated limited diffusion and prolonged release over 14 days, suggesting enhanced retention at the targeted site [80]. Further studies emphasize the effective diffusivity of EVs across various tissue layers. For example, EVs derived from both crude and CD146+ endometrial MSC exhibited similar diffusivity properties, successfully traversing all layers of the meniscus [81]. In addition, CAP-exosomes have shown superior retention in joint environments compared to non-tagged exosome vesicles: following intra-articular injections, CAP-exosomes demonstrated minimal diffusion in vivo, indicating their ability to remain localized at the target site [66].

### 4.3. Dosing and Specs

Optimizing dosing regimens, including injection frequency and intervals, is essential for maximizing therapeutic efficacy. Several studies have explored these parameters using various sources of EVs, concentrations, and dosing schedules.

Qi et al. (2016) [82] highlight that the bioactivity of EVs is highly dependent on their source and microenvironment. They observed that introducing MSC-EVs into sizable bone defects in an osteoporotic rat model promoted both bone regeneration and angiogenesis in a dose-dependent manner. Similarly, Wang et al. (2017) [72] investigated embryonic stem cell-derived EVs in a rat OA model, administering 5 μL of EVs (1 × 10^6^ per joint) intra-articular injections every three days over four weeks. This approach improved cartilage integrity and reduced biomarkers associated with cartilage degradation, including MMPs and aggrecanases. Additionally, the exosomes maintained the chondrocyte phenotype by increasing collagen type II synthesis and decreasing ADAMTS5 expression in the presence of IL-1β.

Chang et al. (2020) [83] examined human adipose-derived stem cell (hASC)-EVs in a monosodium iodoacetate-induced OA model at a dose of 1 × 10^8^ particles per 30 μL volume per joint. Phosphate-buffered saline (PBS) and hyaluronic acid were used as controls under identical conditions for each group. In the subacute arthritis group, EV and HA treatments were applied every 7 days for 3 weeks, starting one week after OA induction. For the chronic OA group, treatments were administered twice weekly for 40 days, beginning two weeks post-OA induction, after notable disease progression was confirmed. The total Mankin score of the hASC-EVs treatment group was significantly lower than that of the PBS or HA treatment group in the acute OA group. The extent of cartilage destruction, chondrocyte hypocellularity, and proteoglycan loss was significantly less compared to the groups treated with PBS or HA. hASC-EV conferred protective effects by preserving the structural integrity of cartilage and maintaining proteoglycan content. According to the same study, destabilization of the medial meniscus (DMM) surgery was performed in a mouse model of PTOA. Five weeks post-surgery, weekly injections of hASC-EVs (1 × 10^8^ particles) or PBS were delivered. Histological analysis showed severe cartilage erosion and proteoglycan loss in the PBS group, whereas the hASC-EV group exhibited minor fibrillations and reduced clefts. The hASC-EV group had a significantly lower OARSI grade and reduced levels of cleaved aggrecan (NITEGE) and MMP-13-positive chondrocytes, markers of cartilage degradation [83].

Liu et al. (2019) [84] compared platelet-rich plasma-derived exosomes (PRP-Exos) to activated PRP (PRP-As) in a rabbit OA model. Four groups were established: (1) control group (no surgery, weekly intra-articular saline injections), (2) OA model group (surgery, weekly intra-articular saline injections), (3) OA + PRP-Exos group (surgery, weekly intra-articular injections of 100 μg/mL PRP-Exos), and (4) OA + PRP-As group (surgery, weekly intra-articular injections of 100 μg/mL PRP-As). Treatments were administered weekly for six weeks post-surgery. PRP-Exos led to increased chondrocyte counts and significantly lower OARSI scores compared to both the OA model and PRP-As groups.

Despite these promising findings, dosing regimens vary considerably, and the therapeutic window for EV therapy remains undefined (Table 3). Differences in EV source, preparation methods, and patient-specific factors may further influence therapeutic outcomes [85]. This variability in effective dosing suggests important gaps that need to be addressed in preparation for translation to humans. A comprehensive review by Jones et al. (2024) emphasizes that factors such as particle concentration, injection frequency, and disease severity may all influence efficacy [78]. Additionally, Rizzo et al. (2023) emphasize that differences in EV preparation methods—including isolation techniques, storage conditions, and administration medium—can significantly alter EV potency [11]. Without a clear therapeutic maximum dose, it remains unclear whether the regimens used in these studies were at, above, or below the optimal dosing threshold. Five clinical trials are currently investigating MSC-EV treatments for early-to-moderate knee OA, all utilizing IA injections [78]. One trial has advanced to dose optimization, assessing clinical outcomes over a one-year period with single injections of 2 × 10^9^, 6 × 10^9^, and 2 × 10^10^ particles per dose using allogeneic umbilical cord MSC-EVs (Trial ID NCT06431152). As clinical trials progress, refining dosing strategies remains a critical focus to balance efficacy and safety in EV-based therapies for OA.

## 5. Logistics

Despite the growing interest in EV-based therapies, several logistical barriers hinder their clinical application. Challenges include incomplete understanding of their mechanisms, difficulty in targeting specific tissues, stability concerns, lack of standardized procedure for creation of EVs, storage, and scalability issues [9]. Furthermore, the generation and purification of EVs are both costly and time-consuming, posing significant barriers to scalability [86]. The heterogeneity of OA pathogenesis further complicates EV therapy development, limiting the identification of universal therapeutic targets and reducing efficacy across diverse patient populations [60]. The lack of standardized guidelines for EV research and a limited number of studies focusing on EV therapies for OA in large animal models compound these challenges, leaving many unanswered questions about their biodistribution and pharmacological behavior [86].

Another major obstacle to clinical translation lies in the post-harvest modification of EVs. Techniques used to load therapeutic cargo into EVs through physical or chemical modifications create both manufacturing and regulatory challenges [62]. Limited scalable isolation methods and inefficient cargo-loading techniques present additional obstacles [53]. Standardization remains a critical issue, as variations in EV composition—stemming from differences in parental cell sources, culture conditions, and isolation methods—pose challenges to reproducibility and therapeutic predictability [78]. This variability necessitates stringent quality control measures to ensure batch-to-batch consistency and hinders large-scale production and global distribution, especially in resource-limited settings [78].

MSC-EV therapies offer some advantages over PRP and MSC-based treatments [78]. Unlike PRP, which faces variability in quality, MSC-EVs are produced under controlled good manufacturing practice (GMP) conditions, ensuring consistency and reducing risks associated with MSC therapy, such as immunorejection and tumorigenicity [78,87]. The use of immortalized cell lines for EV production further reduces batch-to-batch variability, lowers costs, and facilitates easier quality control [78,88]. Though MSC and PRP therapies are more extensively studied, there are many early-stage clinical trials related to EVs for OA treatment. Most focus on evaluating EVs as early biomarkers for monitoring treatment responses, with only a few investigating their therapeutic potential [44]. EVs have an advantage over MSC or other whole-cell treatments, as they may not face the same level of regulatory standards or hurdles [11].

Achieving large-scale production with consistent quality remains a major challenge, as current isolation techniques—such as tangential flow filtration, ultracentrifugation, and size-exclusion chromatography—yield heterogeneous EV populations, complicating reproducibility and scalability [89]. Obtaining large quantities of MSC-derived EVs at an affordable cost remains a major hurdle [44]. Variability in EV production is influenced by factors like parental cell health, culture conditions, and isolation methods. While ultracentrifugation and size-exclusion chromatography work well in research settings, they lack scalability for industrial applications due to low throughput [44]. Alternative methods like tangential flow filtration and precipitation may offer improved efficiency [44,90,91]. Maintaining purity is another challenge, as EV isolation processes risk contamination from proteins, lipids, or infectious agents [44,92,93,94]. Additionally, storage and transportation require temperatures of −80 °C to maintain EV integrity, posing logistical difficulties, particularly in remote areas [44,95]. Addressing these concerns through enhanced purification techniques and room-temperature storage solutions is crucial for clinical viability.

Another concern is the potential for undesirable biomolecular transfer from EVs to recipient cells, particularly with cancer cell-derived EVs, which could have mutagenic effects [44,96,97]. To mitigate these risks, researchers are exploring alternative sources, such as enucleated cells like red blood cells or platelets, for EV production. However, extensive proteomic and genomic analyses are needed to confirm their safety [44].

Efforts to improve EV sustainability for clinical use have led to innovations such as biomimetic EVs, which share some chemical and physical properties with their natural counterparts while allowing for scalable production, increased cargo capacity, and enhanced targeting precision [25]. One example is a hybrid exosome-based nano-sized delivery system, generated by fusing exosomes with liposomes, designed to transport CRISPR/Cas9 plasmids to chondrocytes, thereby reducing extracellular matrix protein degradation [42]. While these advancements in EV technology show promise, substantial research is still required to overcome logistical barriers before EV therapies can be effectively integrated into OA treatment.

## Figures and Tables

**Table 1:** Monoclonal antibodies and cytokine inhibitors under investigation for the treatment of OA.

Drug Name	Targeted Molecule	Mechanism of Action	Stage of Disease in Study	Effects/Findings	Clinical and/or Safety Considerations
**Infliximab**	TNF-α	Monoclonal antibody	Radiographic primary OA of any joint [8] or any hand OA [14]	Reduced pain in OA, improved structural outcomes (structural damage, erosive progression, and presence of active disease) in hand OA but no symptom relief [8,14]	Highlights role of TNF-α in OA-related inflammation and pain [8]
**Adalimumab**	TNF-α	Monoclonal antibody	Radiographic primary OA of any joint [8] or Moderate to severe KOA [15]	Reduced pain, particularly effective when administered intra-articularly [15]	Highlights role of TNF-α in OA-related inflammation and pain, more data needed on long-term safety profile [8,15]
Tanezumab	NGF	Monoclonal antibody	Patients with OA unresponsive to non-opioid medications [16]	Effective in relieving pain in hip and knee OA, with continued relief for up to 56 weeks [16,17,18]	NGF-targeting therapies have inconsistent safety profiles [18,19]
**Fasinumab**	NGF	Monoclonal antibody	Patients with moderate to severe knee or hip OA [20]	Reduced pain and improved function (as measured by WOMAC function score and PGA function score) in hip and knee OA in phase IIb/III trials [13,20]	More research needed due to safety concerns [19,20]
**Fulranumab**	NGF	Monoclonal antibody	Moderate to severe KOA [21] or moderate to severe knee or hip OA [22]	Comparable efficacy to oxycodone in phase II trials, further validated in phase III trials [21,22]	Safety concerns remain inconsistent across studies [19]
**Bevacizumab**	VEGF	Monoclonal antibody	Rabbit model of KOA [23,24,25]	Reduced joint inflammation, synovial proliferation, and cartilage degradation; local intra-articular administration was safer and more effective than systemic administration in animal models [23,24,25]	Most promising monoclonal antibody for slowing OA progression in animal studies [26]
**Canakinumab**	IL-1β	Monoclonal antibody	Patient with knee and hip OA [27]	Reduced need for hip or knee arthroplasty, suggesting disease-modifying potential [27]	IL-1 remains a key cytokine target in OA, but efficacy varies [27]
**AMG 108**	IL-1 receptor	Monoclonal antibody	Patient with KOA [28]	Unsuccessful in alleviating pain in knee OA [28]	Minimal clinical benefit [28]
**Anakinra**	IL-1 receptor	IL-1 receptor antagonist	Patients with KAO [29]	Did not show significant improvement in OA symptoms compared to placebo [17,29]	Demonstrates differences in inflammatory pathways between RA and OA [17,29]
**M6495**	ADAMTS5	Monoclonal antibody	Patients with OA and pain greater than or equal to 40 on WOMAC scale [30]	Under investigation for OA therapy [30]	Emerging target for OA treatment [30]
**IL-1 antibodies**	IL-1	Antibodies against IL-1	Patients diagnosed with KOA [31]	Reduced OA symptoms, improved joint function (as measured with WOMAC and KOOS function scores) [31]	IL-1 inhibitors had safety concerns despite promising results [31]
**IL-1 receptor antibodies**	IL-1 receptor	Antibodies against IL-1 receptor	Patients diagnosed with KOA [31]	Did not show success in OA treatment [31]	Raises questions about effectiveness in different joints [31]

**Table 2:** A Comparison of Various Methods of EV Loading.

Loading Method	Endogenous or Exogenous	Active or Passive	Mechanism	Advantages/Disadvantages
Co-incubation	Endogenous or exogenous	Passive	Cells are incubated with drugs, allowing uptake before EV secretion, or EVs are incubated with drugs, allowing for simple diffusion of cargo into EV [60,63]	Simple, but efficiency depends on drug type and conditions [60,63]
Transfection	Endogenous or Exogenous	Active	Genetic modification of donor cells to express therapeutic cargo [60]	Allows precise cargo control, but has low loading efficiency [60]
Genetic Engineering	Endogenous or Exogenous	Active	Modifies donor cells or EV surface/cargo for precise targeting and therapeutic efficacy [60,61,62,63]	Enables specific therapeutic cargo production, but complex and may have safety concerns [60,61,62,63]
Hypoxic conditions	Endogenous	Active	Cells exposed to low oxygen, influencing EV content [63]	Can enhance therapeutic properties, but is complex and requires specific conditions [60]
Mechanical Stress	Endogenous	Active	Applying physical forces to modify EV composition [60]	Can enhance EV production, but may unpredictably alter cargo composition [60]
3D co-culturing	Endogenous	Active	Culturing cells in a 3D environment to enhance EV loading [60]	More physiologically relevant, but complex to optimize [60]
Endocytosis/Receptor mediated uptake	Endogenous	Passive	Drugs enter cells and get packaged into EVs during biogenesis (can be regulated by cell signaling pathways) [60]	Natural process, but efficiency varies, can be optimized by utilizing receptors for specific targeting [60]
Ultrasonication	Exogenous	Active	Sound waves temporarily disrupt membranes for drug entry [60,61,62,63]	High efficiency, but may damage EVs [60,61,62,63]
Electroporation	Exogenous	Active	Electric fields create pores in membranes for cargo loading [60]	Effective for large molecules, but can cause aggregation [60]
Freeze-thaw cycles	Exogenous	Active	Cycles of freezing and thawing to facilitate cargo loading [60]	Simple, but can lead to cargo degradation [60]

**Table 3:** Summary of Preclinical and Clinical Studies Evaluating Dosing Regimens of EV-Based Therapies in Osteoarthritis.

Study	EV Source	Model	Dose & Route	Frequency/Duration	Key Outcomes
Wang et al., 2017 [72]	Embryonic stem cell-derived EVs	Rat OA model	5 μL (1 × 10^6^ EVs) intra-articular per joint	Every 3 days for 4 weeks	Improved cartilage integrity, ↓ MMPs/aggrecanases, ↑ collagen II, ↓ ADAMTS5 in IL-1β presence
Woo et al., 2020 [83]	hASC EVs	Rat OA (subacute & chronic), mouse PTOA (DMM surgery)	1 × 10^8^ particles in 30 μL per joint	Subacute OA: Weekly x3 (Day 7–28 post-induction) Chronic OA: Twice weekly x6 (Day 14–54 post-induction) DMM: Weekly post-surgery	↓ Mankin and OARSI scores, preserved cartilage structure, ↓ NITEGE & MMP-13-positive chondrocytes
Liu et al., 2019 [84]	PRP-Exos	Rabbit OA model	100 μg/mL intra-articular injections	Weekly x6 post-surgery	↑ chondrocyte count, ↓ OARSI scores vs. OA and PRP-As groups
Clinical Trial (NCT06431152)	Allogeneic umbilical cord MSC-EVs	Knee OA (clinical trial)	2 × 10^9^, 6 × 10^9^, 2 × 10^10^ particles per injection	Single IA injection, 1-year follow-up	Dose optimization trial; clinical outcomes being assessed

## Data Availability

The original contributions presented in the study are included in the article, further inquiries can be directed to the corresponding author.

## Abbreviations

The following abbreviations are used in this manuscript:

OA	Osteoarthritis
IL-1	Interleukin-1
TNF-α	Tumor Necrosis Factor Alpha
EVs	Extracellular Vesicles
MMP-13	Matrix Metalloproteinase-13
ADAMTS5	A Disintegrin and Metalloproteinase with Thrombospondin Motifs 5
KOA	Knee Osteoarthritis
IFP-MSC	Infrapatellar fat pad derived mesenchymal stem cells
IFP	Infrapatellar Fat Pad
IL-1β	Interleukin-1 Beta
IL-6	Interleukin-6
IL-8	Interleukin-8
SP	Substance P
MSC	Mesenchymal Stem/Stromal Cells
CD10	Cluster of Differentiation 10 (Neprilysin)
IA	Intra-Articular
NGF	Nerve Growth Factor
WOMAC	Western Ontario and McMaster Universities Osteoarthritis Index
PGA	Patient Global Assessment
VEGF	Vascular Endothelial Growth Factor
RA	Rheumatoid Arthritis
KOOS	Knee Injury and Osteoarthritis Outcomes Score
PLGA	Poly(lactic-co-glycolic acid)
DSP	Dexamethasone Sodium Phosphate
iPSC	Induced Pluripotent Stem Cells
haMSC/hASC	Human Adipose-Derived Mesenchymal Stem Cells
haMPC	Human Adipose-Derived Mesenchymal Progenitor Cells
IL-10	Interleukin-10
AKI	Acute Kidney Injury
ARDS	Acute Respiratory Distress Syndrome
aCGRP	Antagonist Calcitonin Gene-Related Peptide
AAV	Adeno-Associated Virus
CGRP	Calcitonin Gene-Related Peptide
miRNA	MicroRNA
CAP	Chondrocyte Affinity Peptide
ASO	Antisense Oligonucleotide
sEVs	Small Extracellular Vesicles
FGF18	Fibroblast Growth Factor 18
HAMA	Methacrylic Anhydride-Modified Hyaluronic Acid
ECM	Extracellular Matrix
ESC	Embryonic stem cell-induced
BMSC	Bone Marrow-Derived Mesenchymal Stem Cells
BMP2	Bone Morphogenetic Protein 2
ApoVs	Apoptotic vesicles
PEG	Polyethylene Glycol
PBS	Phosphate-Buffered Saline
DMM	Destabilization of the medial meniscus
PTOA	Post-Traumatic Osteoarthritis
OARSI	Osteoarthritis Research Society International
PRP	Platelet-Rich Plasma
PRP-Exos	Platelet-Rich Plasma-Derived Exosomes
GMP	Good Manufacturing Practice
